# Intraocular Pressure Effects and Mechanism of Action of Topical Versus Sustained-Release Bimatoprost

**DOI:** 10.1167/tvst.8.1.15

**Published:** 2019-01-30

**Authors:** Susan S. Lee, Alexandra Almazan, Sherri Decker, Yan Zhong, Alazar N. Ghebremeskel, Patrick Hughes, Michael R. Robinson, James A. Burke, Robert N. Weinreb

**Affiliations:** 1 Allergan plc, Irvine, CA, USA; 2 Hamilton Glaucoma Center, Shiley Eye Institute and Department of Ophthalmology, University of California San Diego, La Jolla, CA, USA

**Keywords:** bimatoprost, sustained release, intracameral, episcleral venous pressure

## Abstract

**Purpose:**

To assess the intraocular pressure (IOP)-lowering effects of bimatoprost sustained-release implant (BimSR) in normotensive monkeys receiving topical bimatoprost.

**Methods:**

Six eyes from six female, normotensive, cynomolgus monkeys were treated with once-daily topical latanoprost 0.005% plus twice-daily fixed-combination dorzolamide 2%/timolol 0.5%. At week 5, topical latanoprost was switched to once-daily topical bimatoprost 0.03% and twice-daily dorzolamide 2%/timolol 0.5% was continued. At week 8, BimSR 20 μg was administered intracamerally to three eyes and topical therapy was continued in all eyes. At week 12, all topical therapy was discontinued and animals were monitored for another 4 weeks. IOP was measured with a TonoVet rebound tonometer in nonsedated animals weekly for 16 weeks.

**Results:**

Average mean (standard deviation) IOP was 19.8 (1.6) mm Hg at baseline, 15.7 (0.9) mm Hg during treatment with topical latanoprost/dorzolamide/timolol from weeks 1 to 5, and 14.2 (0.5) mm Hg during weeks 6 to 8 after topical latanoprost was switched to topical bimatoprost. After BimSR was added, average mean IOP during weeks 9 to 12 was 10.8 (1.3) mm Hg, a decrease of 3.9 mm Hg compared with the topical-only arm. When topical therapy was discontinued, IOP in BimSR-treated eyes remained below that in unmedicated eyes (15.8 [0.9] vs. 20.2 [0.2] mm Hg at weeks 14–16).

**Conclusions:**

Intracameral BimSR has IOP-lowering effects additive to those of topical bimatoprost, suggesting an additional mechanism of action with intracameral drug delivery.

**Translational Relevance:**

Compared with topical bimatoprost, intracameral BimSR may have an additional mechanism of action of IOP lowering.

## Introduction

Topical medical therapy with prostaglandin analogs (PGAs) is the most common first-line treatment for primary open-angle glaucoma.^1^ Topical PGAs primarily enhance uveoscleral outflow and also increase trabecular outflow facility.^2^–^4^ Although substantial, intraocular pressure (IOP) lowering with PGAs reaches a ceiling due to unknown mechanisms beyond which IOP cannot be lowered any more by increasing the dose. In fact, increasing the dose of topical bimatoprost or latanoprost from once- to twice-daily dosing results in a significant decrease in overall IOP-lowering efficacy.^5^–^8^

**Figure 1:**
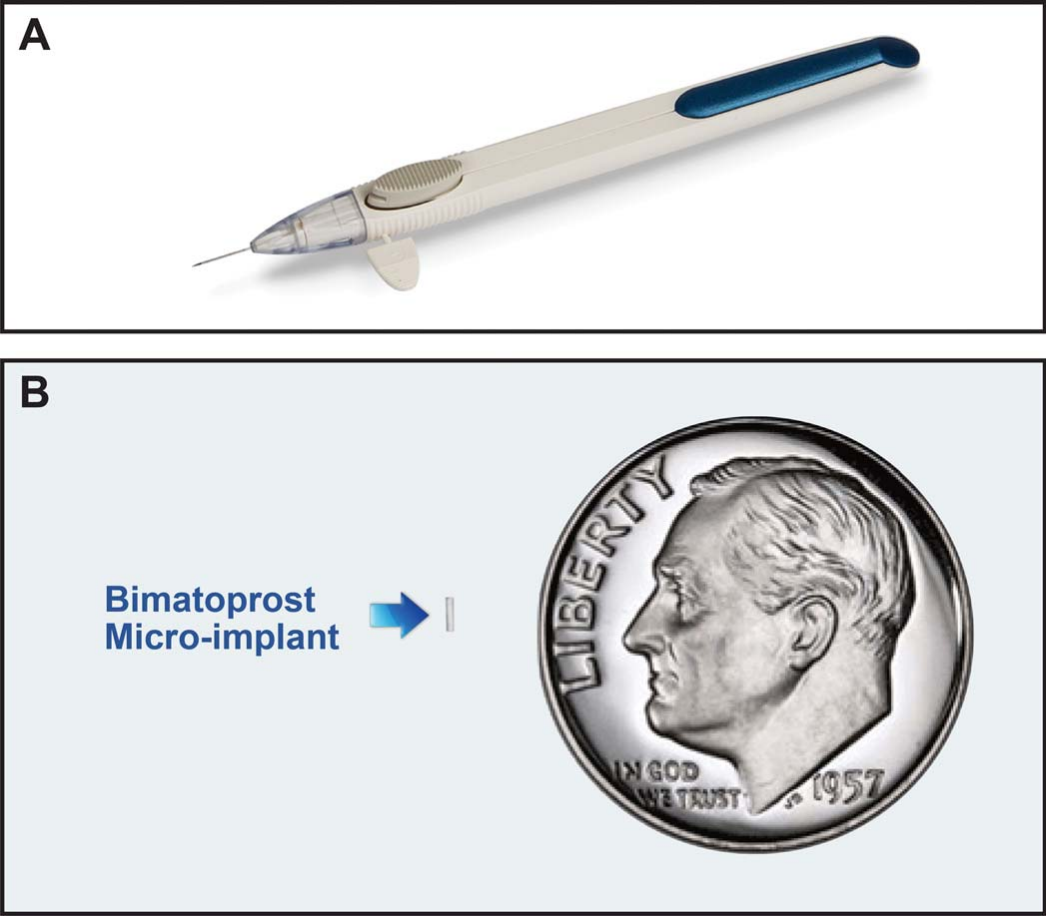
Bimatoprost SR includes (A) a single-use applicator system and (B) a biodegradable micro-implant containing bimatoprost.

Bimatoprost sustained-release intracameral implant (bimatoprost SR) is a biodegradable polymer drug delivery system designed to slowly release bimatoprost into the anterior chamber over a period of 4 to 6 months, while the matrix slowly degrades to inert compounds (Fig. 1).^9^ In a preclinical dose-ranging study, the IOP-lowering efficacy of several doses of bimatoprost SR (8–120 μg) was compared with that of topical bimatoprost 0.03% in ocular normotensive beagle dogs (data on file; Allergan plc, Dublin, Ireland). The magnitude of IOP reduction increased with increasing bimatoprost SR dose throughout the range tested. Moreover, the IOP lowering with bimatoprost SR doses of ≥60 μg exceeded that produced by topical bimatoprost 0.03%, the topical dose that achieves maximal lowering of IOP. This suggested that there may be mechanistic differences between topically and intracamerally administered bimatoprost.

Based on the differences in IOP lowering seen with topical and intracameral dosing of bimatoprost, a difference is hypothesized in the mechanisms of action for each route of administration. If intracameral dosing introduces an additional mechanism of action compared with topical dosing, one can also hypothesize that the combination of intracamerally and topically dosed bimatoprost should achieve an IOP reduction greater than topical dosing alone. We tested the hypothesis that intracameral bimatoprost, delivered with bimatoprost SR, produces additional IOP lowering when added to a topical regimen.

## Methods

This study was performed at Allergan plc under an approved Animal Care and Use Committee protocol. All procedures were in compliance with Animal Welfare Act Regulations (9 CFR 3) and routine veterinary care was performed in accordance with the Animal Welfare Act, the Guide for the Care and Use of Laboratory Animals, the Office of Laboratory Animal Welfare, and the ARVO Statement for the Use of Animals in Ophthalmic and Vision Research.

Six female, normotensive, drug-naïve cynomolgus monkeys weighing 2.6 to 3.2 kg were studied. To reduce the chance that the IOP measurements could be confounded by the effects of anesthesia, all animals were chair-trained for awake IOP examinations for 3 months before the study so that IOP measurements could be performed without topical or general anesthesia.

**Figure 2:**
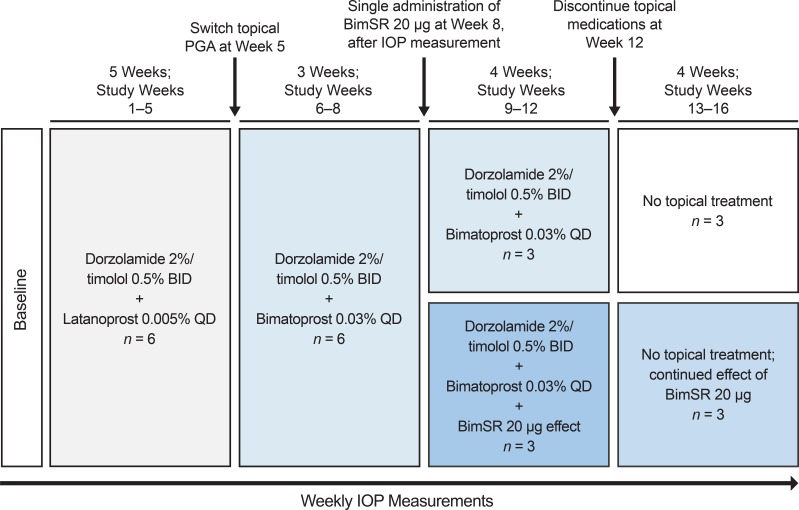
Study design. BID, twice daily; BimSR, bimatoprost sustained-release implant (bimatoprost SR); FC, fixed-combination; QD, once daily.

The left eye was designated as the study eye. In all animals, the study eye was treated with topical latanoprost 0.005% (Xalatan; Pfizer Inc., New York, NY) once daily plus topical fixed-combination dorzolamide 2%/timolol 0.5% (Cosopt; Merck & Co., Kenilworth, NJ) twice daily for 5 weeks (Fig. 2). A multiple-drug topical regimen of PGA, carbonic anhydrase inhibitor, and beta-blocker frequently is used in glaucoma patients as a maximal tolerated medical therapy^10^ and was used in the animals in an attempt to reduce IOP as much as possible by maximally increasing aqueous outflow and decreasing aqueous production. To assess the differences in efficacy between monkey and humans, at week 5 after IOP was measured, topical latanoprost was switched to topical bimatoprost 0.03% (Lumigan; Allergan plc) once daily while dorzolamide/timolol twice daily was continued. An additional 1 mm Hg of mean IOP lowering was observed 1 week later (Table 1), confirming that topical bimatoprost exhibited a similar difference in efficacy compared with latanoprost as when used in humans, which increases confidence in the translatability of the study results. The animals were continued on the regimen of topical bimatoprost plus dorzolamide/timolol. All doses of topical latanoprost and bimatoprost were given 5 minutes after the evening dose of dorzolamide/timolol. Topical therapy was continued in all eyes through week 12, when the topical therapy was discontinued in all eyes after IOP measurement. The animals then were monitored for an additional 4 weeks.

**Table 1:** Mean IOP (± SEM) at Key Time Points, mm Hg

Time and Treatment	All Animals, *n* = 6	BimSR Added to Topical Triple Therapy, *n* = 3	No Implant Added to Topical Triple Therapy, *n* = 3
Baseline: before any treatment	19.8 ± 0.7	—	—
Week 1: 1 week after initiating topical dorzolamide/timolol/latanoprost triple therapy	15.8 ± 1.0*	—	—
Week 6: 1 week after switch to topical dorzolamide/timolol/bimatoprost triple therapy	14.8 ± 1.1*	—	—
Week 9: 1 week after addition of BimSR or no implant to topical dorzolamide/timolol/bimatoprost triple therapy	—	11.0 ± 0.5*^,^†	14.5 ± 1.3*
Week 12: 4 weeks after addition of BimSR or no implant to topical dorzolamide/timolol/bimatoprost triple therapy	—	9.5 ± 0.6*^,^†	14.5 ± 1.8*
Week 16: 4 weeks after all topical therapy stopped	—	16.5 ± 1.3*^,^†	20.0 ± 1.5

BimSR, bimatoprost sustained-release implant (bimatoprost SR).

* *P* < 0.005 vs. baseline,

† *P* ≤ 0.012 vs. no implant added to topical triple therapy.

At week 8, bimatoprost SR 20 μg was administered to the study eyes of three animals following the IOP measurement. Before dosing with bimatoprost SR, each animal was sedated with intramuscular ketamine (15 mg/kg) and atropine (0.022 mg/kg) and the eye was anesthetized with topical proparacaine hydrochloride 0.5%. Povidone iodine 5% drops were applied preoperatively for 2 minutes and the eye was rinsed with balanced salt solution. A lid speculum was placed and the eye was visualized through an operating microscope. Bimatoprost SR was inserted into the anterior chamber using a single-use applicator equipped with a 27-gauge needle. The needle was introduced just anterior to the insertion of the conjunctiva at the limbus through the clear cornea in the superotemporal quadrant using countertraction with a tooth forceps lightly grasping the conjunctiva near the limbus. Topical gatifloxacin 0.3% eye drops were placed in the eye postoperatively.

A TonoVet veterinary rebound tonometer (Icare USA, Raleigh, NC) was used to measure IOP on calm (pole and collar handling system), fully conscious, nonsedated animals. The IOP was measured at the same time of day throughout the study (10 AM ± 1 hour). The baseline IOP measurement was taken on the day before topical dosing was initiated, and after initiation of topical dosing, IOP was measured every 7 days through week 16. The morning dose of dorzolamide/timolol was administered at 8 AM ± 1 hour, and IOP was measured 2 hours later (peak effect).

A mixed-model repeated-measures method was used for statistical analysis: IOP was the response variable, and the dose group (topical therapy only or topical therapy + bimatoprost SR), time, and dose group by time interaction were treated as fixed effects. The data were fitted to the model, and comparisons with baseline and between dose groups were made based on the fitted model. All comparisons reported were from the same model; no adjustment was made for multiple comparisons due to the small sample size.

## Results

**Figure 3:**
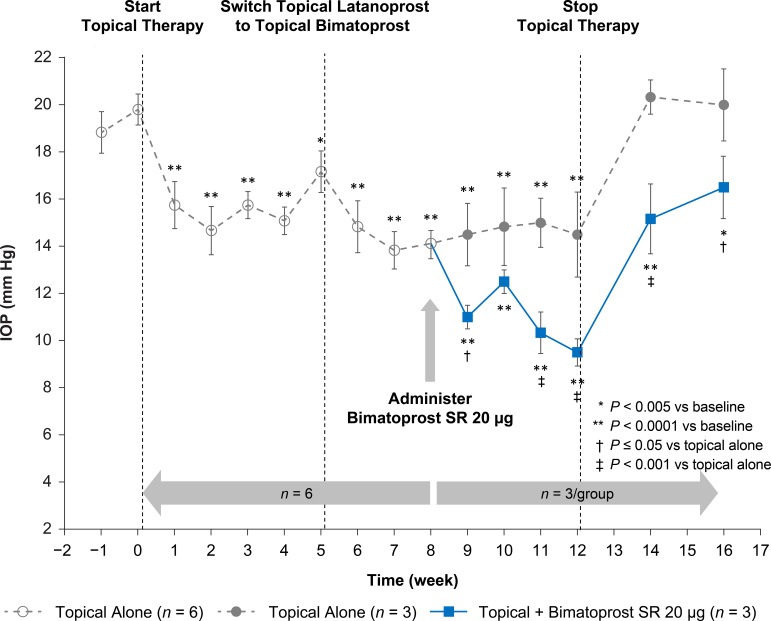
Effect of bimatoprost SR and topical ocular hypotensive therapy on mean IOP in normotensive monkeys. Topical therapy consisted of dorzolamide 2%/timolol 0.5% twice daily plus latanoprost once daily in the evening (weeks 0–5) or dorzolamide 2%/timolol 0.5% twice daily plus bimatoprost once daily in the evening (weeks 5–12). IOP was measured 2 hours after morning topical dosing, at peak effect. IOP values shown are mean ± SEM.

Table 1 shows mean values of IOP observed at key time points in the study, and Table 2 shows estimates of IOP changes from baseline at each postbaseline time point in the repeated-measures mixed-effects model. Mean (± standard deviation) baseline IOP was 19.8 ± 1.6 mm Hg, and during treatment with topical latanoprost/dorzolamide/timolol therapy from weeks 1 to 5, the average mean IOP was 15.7 ± 0.9 mm Hg (*P* < 0.005 vs. baseline at all time points; Fig. 3). When latanoprost in the triple combination topical therapy was switched to bimatoprost at week 5, there was an additional decrease in average mean IOP to 14.2 ± 0.5 mm Hg (*P* < 0.0001 vs. baseline at all time points) through week 8. The average mean IOP reduction of the topical combination with bimatoprost (weeks 6–8) was 1.4 mm Hg greater than with latanoprost (weeks 1–5; *P* = 0.0185). When bimatoprost SR was added to this triple combination in half of the monkeys (*n* = 3), there was an additional, statistically significant decrease in average mean IOP during weeks 9–12 to 10.8 ± 1.3 mm Hg (*P* ≤ 0.01 vs. topical alone at weeks 9, 11, and 12), which was an additional decrease of 3.9 mm Hg compared to the topical-only group. When topical therapy was discontinued, IOP increased, but the IOP in eyes treated with bimatoprost SR remained below that in unmedicated eyes at weeks 14 (*P* = 0.0002) and 16 (*P* = 0.0117).

**Table 2:** Estimates of IOP Changes From Baseline in the Mixed-Model Repeated-Measures Analysis, mm Hg

Treatment	Week	Estimate of IOP Change From Baseline	Limits of 95% Confidence Interval
Topical dorz/tim + lat, *n* = 6	1	−4.06	−5.26, −2.85
Topical dorz/tim + lat, *n* = 6	2	−5.14	−6.34, −3.94
Topical dorz/tim + lat, *n* = 6	3	−4.06	−5.26, −2.85
Topical dorz/tim + lat, *n* = 6	4	−4.72	−5.93, −3.52
Topical dorz/tim + lat, *n* = 6	5	−2.64	−3.84, −1.44
Topical dorz/tim + bim, *n* = 6	6	−4.97	−6.18, −3.77
Topical dorz/tim + bim, *n* = 6	7	−5.97	−7.18, −4.77
Topical dorz/tim + bim, *n* = 6	8	−5.72	−6.93, −4.52
Bimatoprost SR administered at week 8 after IOP measurement			
Topical dorz/tim + bim, *n* = 3	9	−8.79	−10.52, −7.05
Topical dorz/tim + bim, *n* = 3	10	−7.29	−9.02, −5.55
Topical dorz/tim + bim, *n* = 3	11	−9.45	−11.19, −7.71
Topical dorz/tim + bim, *n* = 3	12	−10.29	−12.02, −8.55
No topical therapy, *n* = 3	14	−4.62	−6.36, −2.88
No topical therapy, *n* = 3	16	−3.29	−5.02, −1.55
No Bimatoprost SR at week 8			
Topical dorz/tim + bim, *n* = 3	9	−5.33	−7.07, −3.59
Topical dorz/tim + bim, *n* = 3	10	−4.99	−6.73, −3.25
Topical dorz/tim + bim, *n* = 3	11	−4.83	−6.57, −3.09
Topical dorz/tim + bim, *n* = 3	12	−5.33	−7.07, −3.59
No topical therapy, *n* = 3	14	+0.51	−1.23, +2.25
No topical therapy, *n* = 3	16	+0.17	−1.57, +1.91

Bim, bimatoprost 0.03% once daily; Bimatoprost SR, bimatoprost sustained-release implant 20 μg; dorz/tim, fixed-combination dorzolamide 2%/timolol 0.5% twice daily; lat, latanoprost 0.005% once daily.

No intraoperative or postoperative complications of bimatoprost SR administration were observed. In addition, no adverse effects were documented in eye examinations performed before the IOP measurements.

## Discussion

This was the first study to evaluate the additive IOP-lowering effects of bimatoprost SR with topical IOP-lowering medications. The results showed that addition of intracameral bimatoprost SR provided further IOP lowering in monkeys already receiving topically administered bimatoprost 0.03% once daily. A previous study in cynomolgus monkeys demonstrated that the clinical dose of topical bimatoprost (9 μg, the dose in a 30 μL drop of bimatoprost 0.03%) reduces IOP at least as effectively as a dose 3 times higher (27 μg), and administering the clinical dose of topical bimatoprost twice daily results in less IOP lowering than once-daily dosing.^11^ This suggests that in monkeys, as in humans,^5^,^6^,^12^ once-daily administration of the bimatoprost 0.03% formulation produces the maximal IOP lowering achievable with topical bimatoprost administration. Thus, the additional IOP lowering produced by bimatoprost SR in monkeys treated with once-daily topical bimatoprost 0.03% suggests that intracameral PGA dosing may have an additional mechanism of action for IOP lowering beyond the expected increases in uveoscleral outflow and possibly outflow facility. Since the eyes were on two potent aqueous humor production suppressants (i.e., dorzolamide/timolol), the additional mechanism of action was not likely due to additional aqueous production suppression.

An unpublished study in beagle dogs showed that the IOP lowering produced by bimatoprost SR is dose-dependent and at higher doses exceeds that produced by topical bimatoprost administration (Allergan plc; data on file). Similarly, in a phase 1/2 study in human subjects with glaucoma, intracameral administration of bimatoprost with bimatoprost SR produced dose-dependent IOP lowering. Moreover, at the higher dose strengths of the implant, the mean IOP reduction at early time points in study eyes was incrementally greater than the mean IOP reduction in fellow eyes treated with once-daily topical bimatoprost 0.03%.^9^ These findings also are consistent with the hypothesis that intracameral delivery of bimatoprost with bimatoprost SR has an additional mechanism of IOP lowering compared with topical dosing.

The 20-μg dose strength of bimatoprost SR used in this study in monkeys was the same as the highest dose strength of bimatoprost used in the phase 1/2 clinical trial.^9^ Reported interim results of the clinical study showed that a single administration of implant controlled IOP in 71% of patients with glaucoma for up to 6 months.^9^ Consistent with the clinical findings, in our study significant IOP lowering was produced by the implant throughout the follow-up period (up to 8 weeks after administration). From weeks 8–12, bimatoprost SR reduced the IOP in eyes already treated with topical timolol/dorzolamide/bimatoprost, and after the topical regimen was stopped at week 12, bimatoprost SR continued to reduce IOP relative to the baseline IOP through week 16.

A possible explanation for the additional IOP lowering produced by bimatoprost SR when added to a topical regimen including bimatoprost is that the implant produces much higher concentrations of bimatoprost in the target tissues within the eye than is possible with topical dosing, as has been demonstrated in a study in dogs,^13^ and these higher concentrations may be able to enhance uveoscleral outflow and/or outflow facility. The ability of a topically applied drug to reach the intraocular tissues controlling aqueous outflow is limited by its ability to penetrate the cornea or conjunctiva/sclera. Intracameral drug delivery removes these barriers to drug penetration to intraocular target tissues. This explanation does not seem consistent with the inability of higher topical concentrations of bimatoprost to improve IOP lowering, since increasing the concentration of bimatoprost applied topically also would be expected to increase intraocular drug concentrations. However, the reasons for the ceiling effect for IOP lowering with increased concentrations of topical PGAs are not well understood, and it remains possible that the location or kinetics of drug delivery with the implant, or the very high drug concentrations achieved at target tissues after intracameral administration, results in enhancement of uveoscleral outflow and/or outflow facility and improved IOP lowering.

**Figure 4:**
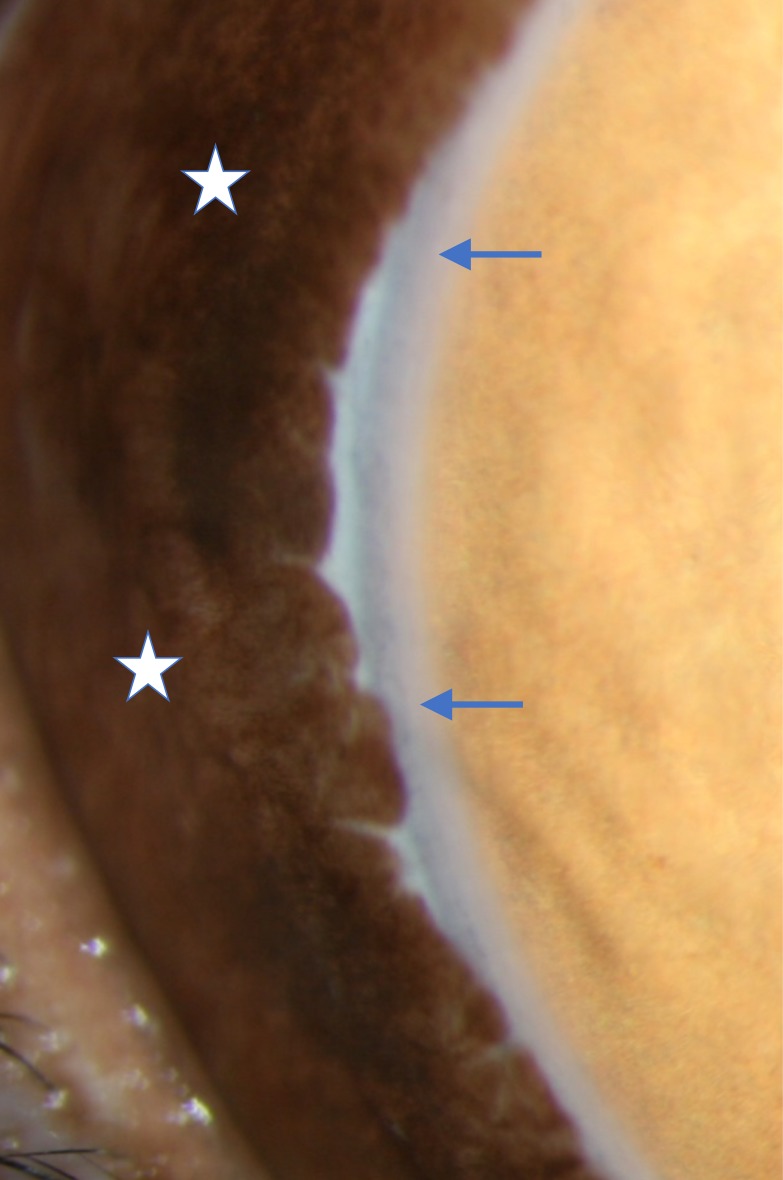
Photograph showing dense perilimbal pigmentation of an eye in a cynomolgus monkey. Blue arrows, corneal limbus; white stars, perilimbal pigmentation.

A second possible explanation for the additive IOP lowering produced by bimatoprost SR in monkeys treated with topical medications is that its mechanism of IOP lowering involves a decrease in episcleral venous pressure (EVP), as well as an increase in uveoscleral outflow and outflow facility. This possibility was suggested by a previous study in dogs that showed that intracameral delivery of bimatoprost SR led to a significant, sustained decrease in EVP following a transient increase in EVP.^14^ In contrast, no decrease in EVP occurred in dogs after topical administration of latanoprost^15^ or bimatoprost (Allergan plc, data on file). Preclinical studies of IOP regulation in dogs and monkeys provide useful information relevant to human physiology, because the anatomy and function of aqueous outflow systems are similar in humans, monkeys, and dogs.^16^ Dogs have a venous plexus instead of a true Schlemm's canal, but aqueous collector vessels are found in dogs, humans, and monkeys.^16^ The flow of aqueous from the collector vessels into the episcleral vasculature, and consequently the IOP, is influenced by the EVP.^17^,^18^ EVP can be measured invasively by cannulation of the episcleral vein or noninvasively using a pressure chamber (episcleral venomanometer) that measures the pressure required to constrict the vein to a predetermined endpoint.^19^ In animal studies, these methods produce comparable measurements of EVP.^19^ Regardless of the method used for EVP measurement, EVP has been reported to be similar across species. EVP measured with a venomanometer has been reported to be approximately 10 mm Hg in normotensive beagle dogs^14^ and ranged from approximately 8 to 11 mm Hg in normal human subjects.^18^ It is not possible to measure EVP noninvasively in cynomolgus monkeys, because dense perilimbal pigmentation just 4 to 6 mm from the limbus impedes visualization of the episcleral outflow vessels (Fig. 4), and the anatomy of the eye, deeply inset in the tight orbit, prevents applanation of the episcleral vessels with the commercially available venomanometer. However, mean EVP measured in cynomolgus monkeys by direct cannulation was reported to be 10.4 mm Hg,^20^ similar to the EVP measured noninvasively in humans and dogs.

We chose to do the study in cynomolgus monkeys because of the close relationship of the species to humans. Monkey eyes are similar to human eyes in most respects, and importantly, topical medications used to lower IOP in humans with glaucoma have similar effects on aqueous dynamics and demonstrate similar IOP-lowering effects in monkeys. Furthermore, topical PGAs have been shown to produce similar percentage IOP reductions in normotensive monkey eyes as in laser-induced ocular hypertensive monkey eyes.^21^,^22^ We also have observed a low-grade chronic anterior uveitis in the laser-induced ocular hypertensive monkey eye model (Tsai S, et al. *IOVS*. 2011;52:ARVO E-Abstract 2435), and the degree to which this could confound the IOP measurements in this implant study was not known. Therefore, normotensive monkeys were used in this study as a more appropriate model to evaluate drug effects on IOP that are translatable to human eyes with glaucoma.

In conclusion, our study results suggest that bimatoprost SR may have an additional IOP-lowering mechanism of action that differentiates it from topical PGAs. This incremental additional IOP lowering may be related to EVP reduction, but other explanations are possible. Additional mechanism of action studies are needed to further explore reasons for differential effects of topically and intracamerally administered bimatoprost in animals and humans.
